# Risk Factors for Alanine Aminotransferase Elevations in a Prospective Cohort of HIV-Infected Tanzanian Adults Initiating Antiretroviral Therapy

**DOI:** 10.1177/2325958219884939

**Published:** 2019-10-31

**Authors:** Sabina F. Mugusi, David Sando, Ferdinand M. Mugusi, Claudia Hawkins, Said Aboud, Wafaie W. Fawzi, Christopher R. Sudfeld

**Affiliations:** 1 Department of Clinical Pharmacology, School of Medicine, Muhimbili University of Health and Allied Sciences, Dar es Salaam, Tanzania; 2 Department of Global Health and Population, Harvard T.H. Chan School of Public Health, Boston, MA, USA; 3 Department of Internal Medicine, School of Medicine, Muhimbili University of Health and Allied Sciences, Dar es Salaam, Tanzania; 4 Center for Global Health, Division of Infectious Diseases, Northwestern University Feinberg School of Medicine, Chicago, IL, USA; 5 Department of Microbiology and Immunology, School of Medicine, Muhimbili University of Health and Allied Sciences, Dar es Salaam, Tanzania

**Keywords:** HIV, antiretroviral, hepatitis, ALT, liver, hepatotoxicity

## Abstract

**Introduction:**

Serum alanine aminotransferase (ALT) elevations are common among HIV-infected patients on combination antiretroviral therapy (cART).

**Approach:**

We conducted a prospective cohort study of 3023 HIV-infected Tanzanian adults initiating cART. We assessed risk factors for mild/moderate ALT elevations >40 IU/L and severe ALT elevations >200 IU/L.

**Results:**

We found that over a median follow-up of 32.5 months (interquartile range: 19.4-41.5), 44.8% of participants had at least 1 incident ALT elevation >40 IU/L of which 50.1% were persistent elevations. Risk factors for incident ALT elevation >40 IU/L included male sex, CD4 count <100 cells/μL, d4T+3TC+NVP cART, and triglycerides ≥150 mg/dL (*P* values <.05). Hepatitis B coinfection and alcohol consumption increased the risk of severe ALT elevations >200 IU/L (*P* values: <.05).

**Conclusion:**

Incident mild and moderate ALT elevations are common among Tanzanians initiating cART, and the clinical and demographic information can identify patients at increased risk.

**What Do We Already Know about This Topic?**

Liver enzyme elevations are common among HIV-infected patients initiating combination antiretroviral therapy due to multiple factors, such as direct hepatocyte inflammation, the antiretroviral used, comorbidities, and drug–drug interactions.

**How Does Your Research Contribute to the Field?**

Analysis from this large longitudinal cohort study provides information on incident and sustained alanine aminotransferase (ALT) elevations as well as the risk factors associated with these elevations among Tanzanian HIV-infected patients initiating antiretroviral therapy.

**What Are Your Research’s Implications toward Theory, Practice, or Policy?**

Patients who are identified to be at a higher risk of having ALT elevations based on clinical and demographic information should be closely monitored by clinicians.

## Introduction

Serum alanine aminotransferase (ALT) is generally used for evaluating liver function and is a surrogate marker of liver injury.^1,2^ Studies among HIV-infected adults have found that men are more likely to have ALT elevations compared to women. Levels of ALT have been associated with high body mass index (BMI), dyslipidemia, and hypertension.^3,4^ Liver enzyme elevations are frequent among HIV-infected patients due to multiple factors, including direct inflammation in hepatocytes, use of antiretroviral drugs, coinfection with hepatitis, opportunistic infections, alcoholism, nonalcoholic steatohepatitis (NASH), and toxicities related to nonantiretroviral drugs (eg, antimicrobials, lipid medications).^5-8^ Liver disease is a leading cause of non–AIDS-related mortality in high-income settings, and the relative contribution may be growing in resource-limited settings due to expanding combination antiretroviral therapy (cART) coverage as programs transition to test and treat strategies.^9^

A number of risk factors for liver enzyme abnormalities among HIV-infected adults on cART have been identified, primarily from studies conducted in high-income settings. Most of the antiretroviral drug classes have been associated with liver enzyme abnormalities.^10,11^ Hepatotoxicity related to the use of nucleoside reverse transcriptase inhibitors (NRTIs) and non-NRTIs (NNRTIs), especially stavudine (d4T), zidovudine (AZT), didanosine, and nevirapine (NVP), has been widely reported.^12,13^ Hepatitis B and C coinfection is also well documented to increase the risk of hepatotoxicity.^14^ In addition, treatment of tuberculosis and other opportunistic infections can also increase the risk of liver abnormalities, since antimycobacterial and antimicrobial drugs can induce liver damage and potently interact with cytochrome P450 enzymes.^15^ Nevertheless, few longitudinal studies have examined the risk factors for incident ALT elevations in the context of sub-Saharan Africa.

In order to address this research gap, we present a prospective cohort study of Tanzanian adults initiating cART. We examine risk factors for incident ALT elevations of >40 IU/L and >200 IU/L as well as sustained liver enzyme abnormalities, since ALT elevations can be transient. This study intends to identify patient characteristics at cART initiation that are associated with high risk of ALT elevation to better guide clinical care.

## Methods

This prospective cohort study included HIV-infected adults initiating cART who were enrolled in a randomized, double-blind trial that assessed the effect of daily oral supplements of vitamins B complex, C, and E in multiple versus single levels recommended dietary allowance (RDA) on HIV disease progression in Dar es Salaam, Tanzania, during 2006 to 2010.^16^ Briefly, individuals were eligible for the trial if they were aged ≥18 years, HIV infected, initiating ART at enrollment, and intended to stay in Dar es Salaam for at least 2 years. Women who were pregnant or lactating were excluded. Following the World Health Organization (WHO) and Tanzania HIV/AIDS treatment guidelines at the time, newly diagnosed HIV-infected patients and eligible to start cART included were those with WHO clinical stage IV disease or CD4 count of <200 cells/μL or with WHO clinical stage III disease and CD4 count of <350 cells/μL.^17,18^ During the period of study, the first-line drug combinations included d4T, lamivudine (3TC), NVP, AZT, and efavirenz (EFV). Stavudine was switched to AZT for individuals with peripheral neuropathy or for those who did not tolerate d4T. Nevirapine was switched to EFV in patients who could not tolerate NVP. As a result, 4 different ART regimens were used in trial: (1) d4T+3TC+NVP, (2) d4T+3TC+EFV, (3) AZT+3TC+NVP, and (4) AZT+3TC+EFV. Co-trimoxazole prophylaxis was provided for individuals whose CD4 counts were <200 cells/µL. Eligible patients were randomized to receive either the standard RDA or the multiple RDA (2-21 times the RDA for the vitamin B, 2 times the RDA for vitamin E, and 6 times the RDA for vitamin C).^16^

At enrollment, a standardized questionnaire was used to collect sociodemographic characteristics and past medical history, followed by a full clinical examination. Height and weight measurements were taken at baseline and all follow-up visits using standardized procedures and calibrated instruments. Data on alcohol use were collected at baseline using yes-and-no responses; however, the data on the frequency and amount consumed were not collected. The study participants were seen monthly per the standard of care using the program guidelines for clinical assessment and antiretroviral drug pick-up. Laboratory investigations were performed at baseline and every 4 months in accordance with the routine tests recommended for clinical care per the country/program guidelines. Absolute CD4 count (FACS Calibur flow cytometer, Becton Dickinson, San Jose, California), a complete blood count (AcT5 Diff AL analyzer, Beckman Coulter, Miami, Florida), and HIV viral load (Cobas Amplicor HIV-1 Monitor test version 1.5, Roche Diagnostics Systems) were assessed at and every 4 months thereafter for the study duration. Hepatitis B surface antigen and hepatitis C antibody tests were conducted at enrollment and as indicated thereafter using rapid tests (ACON Laboratories Inc Indianapolis, Indiana).

Serum samples for ALT assessment were collected at randomization and every 4 months thereafter. Blood for ALT analysis was collected in redtop vacutainer tubes and separated into serum on the collection by centrifugation at 1600 g for 10 minutes and stored up to 7 days at 2°C to 8°C. The concentrations of ALT were measured using Cobas Integra 400 plus analyzer by Roche Diagnostics System. The elevation in ALT was defined using the Division of AIDS criteria for Grading the Severity of Adult Adverse Events.^19^ Elevated ALT defined as >40 IU/L or >1× the upper limit of normal (ULN) was the primary outcome of this analysis. Sustained ALT elevation was defined as ALT >40 IU/L at 2 or more consecutive ALT assessments.^16^ In addition, we defined severe ALT elevations as patients having ALT levels >200 IU/L or >5× ULN.

### Statistical Analysis

We examined cART initiation risk factors for incident ALT elevations using Cox proportional hazard models. We analyze time to first ALT elevation >40 IU/L, first elevation of sustained ALT >40 IU/L, and time to first ALT >200 IU/L. Individuals with the outcome of interest at the baseline cART initiation visit were excluded from each analysis. We examined the following predictors, assessed at cART initiation, for elevated ALT: sex (male, female), age (18-30, 30-45, >45 years), WHO clinical stage (I or II, III, and IV), CD4 count (<100, 100-199, ≥200 cells/μL), HIV viral load (<100 000, 100 000-1 000 000, >1 000 000 copies/mL), BMI (<18.5 kg/m^2^ versus ≥18.5 kg/m^2^), hemoglobin level (<8.5 g/dL versus ≥8.5 g/dL), cholesterol (<200 mg/dL versus ≥200 mg/dL), triglycerides (<150 mg/dL versus ≥150 mg/dL), and multivitamin (single RDA, multiple RDA). Multivariate models included all the variables used in the univariate analysis. Missing data for covariates were retained in the analysis, using the missing indicator method. All *P* values were 2 sided, with *P* values of <.05 considered statistically significant. Statistical analyses were performed using Stata version 15 (StataCorp, College Station, Texas).

### Ethical Approval and Informed Consent

Written informed consent was obtained from all participants included in the study. The study protocol was approved by the institutional review boards of the Harvard School of Public Health (IRB12981), Muhimbili University of Health and Allied Sciences (MU/DRP/AEC/Vol.XVI/164), Tanzania Food and Drugs Authority (CD/TFDA.226/6), and the National Health Research Ethics Sub-Committee (NIMR/HQ/R.8a/Vol. IX/432).

## Results

A total of 3418 patients were recruited into the trial, of which 3023 (88.4%) patients had ALT measured at baseline and at least once during the follow-up period and are included in this analysis. The median follow-up time for the cohort was 32.5 months (interquartile range [IQR]: 19.4-41.5). Table 1 summarizes the baseline sociodemographic and clinical characteristics of the study cohort. The majority of the cohort was female (68.3%) and between the ages of 31 and 45 years (65.0%). Forty-one percent of the patients were severely immunocompromised with CD4 counts below 100 cells/μL at cART initiation, with over two-thirds of the patients having viremia of >100 000 copies/mL. Comorbidity with tuberculosis was reported in 1.1% of the study population. Additionally, 6% of patients were hepatitis B coinfected while 2% were anti-hepatitis C positive. In addition, 11.8% of patients had high cholesterol (≥200 mg/dL) and 21% with high triglycerides (≥150 mg/dL). Two-thirds of the patients were placed on the first-line d4T+3TC+NVP cART regimen.

**Table 1.** Baseline Sociodemographic and Clinical Characteristics of ALT Study Cohort (n = 3023).

Characteristic	n (%)
Sex	
Females	2066 (68.3)
Males	957 (31.7)
Age, years	
≤30	463 (15.3)
31-45	1962 (65.0)
>45	592 (19.7)
WHO HIV disease stage	
I or II	651 (21.9)
III	1802 (60.6)
IV	340 (11.5)
CD4 count, cells/μL	
<100	1213 (41.3)
100-200	1113 (37.9)
>200	608 (20.8)
HIV viral load, copies/mL	
<100 000	386 (29.8)
100 000-1 000 000	829 (64.0)
>1 000 000	80 (6.2)
Body mass index, kg/m^2^	
<18.5	706 (25.1)
18.5-25	1660 (58.9)
>25	449 (16.0)
Hemoglobin, g/dL	
≥8.5	2279 (78.1)
<8.5-severe anemia	638 (21.9)
cART regimen	
d4T+3TC+NVP	1785 (66.6)
d4T+3TC+EFV	302 (12.3)
AZT+3TC+NVP	249 (9.3)
AZT+3TC+EFV	344 (12.8)
Self-reported alcohol use	
No	2744 (98.1)
Yes	52 (1.9)
Tuberculosis coinfection	
No	2991 (98.9)
Yes	32 (1.1)
Hepatitis B	
Negative	1927 (93.8)
Positive	127 (6.2)
Hepatitis C	
Negative	1593 (98.3)
Positive	27 (1.7)
Total cholesterol, mg/dL	
<200	2050 (88.2)
≥200	273 (11.8)
Triglycerides, mg/dL	
<150	1795 (79.1)
≥150	473 (20.9)
Randomized multivitamin regimen	
Single RDA	1522 (50.4)
Multiple RDA	1501 (49.6)
ALT concentration, IU/L	
≤40	2667 (88.2)
41-200	353 (11.7)
>200	3 (0.1)

Abbreviations: ALT, alanine aminotransferase; AZT, zidovudine; cART, combination antiretroviral therapy; d4T, stavudine; EFV, efavirenz; NVP, nevirapine; RDA, recommended dietary allowances; WHO, World Health Organization; 3TC, lamivudine.

At baseline, 2667 (88.2%) patients had normal ALT concentration ≤40 IU/L, 353 (11.7%) had mild or moderate ALT elevations of 41 to 200 IU/L, and 3 (0.1%) patients had a severe ALT elevation >200 IU/L at randomization. A total of 21 148 follow-up ALT measurements were done with a median of 8 (IQR: 6-11) ALT tests per patient during the follow-up period. During follow-up, 23.2% of patient had an incident ALT elevation >40 IU/L and 23.3% had an incident sustained ALT elevation >40 IU/L. The median time to first ALT elevation >40 IU/L was 140 days (IQR: 58-338). A total of 75 (2.5%) patients had an incident severe ALT elevation >200 IU/L assessed at a median time of 123 days postrandomization (IQR: 30-428), where 22 (29%) of these patients had these elevations within the first month of initiating cART.

Table 2 presents univariate and multivariate cART initiation risk factor analyses for incident mild and moderate ALT elevations >40 IU/L. In univariate models, male sex, CD4 count <100 cells/μL, d4T+3TC+NVP regimen, hepatitis C coinfection, and high triglyceride levels (≥150 mg/dL) were associated with incident ALT elevations >40 IU (*P* values <.05). In multivariate analysis, males remained at increased risk of incident ALT >40 IU/L when compared to females (hazard ratio [HR]: 1.44; 95% confidence interval [CI], 1.27-1.64; *P* value: <.001). Patients initiated on d4T+3TC+NVP had 1.44 (95% CI, 1.17-1.76; *P* < .001) times the risk of incident ALT >40 when compared to those receiving AZT+3TC+EFV. Patients with CD4 counts of 100 to 200 cells/μL and >200 cells/μL at cART initiation had 19% (95% CI, 8%-29%) and 26% (95% CI, 13%-37%) lower risk of developing ALT >40 IU/L when compared to those who had CD4count <100 cells/μL, respectively. Individuals with serum triglyceride concentration >150 mg/dL (HR: 1.31; 95% CI, 1.12-1.54; *P* value: .01) and those randomized to multiple RDA multivitamins (HR: 1.41; 95% CI, 1.26-1.58; *P* value: <.001) were also at increased risk. Patients who were hepatitis C positive appeared to be at higher risk of incident ALT >40; however, the results did not reach statistical significance (HR: 1.64; 95% CI, 0.99-2.71).

In terms of cART initiation predictors for sustained ALT elevations >40 IU/L, we found WHO HIV stage III disease (HR as compared to stage I or II: 0.76; 95% CI, 0.63-0.93; *P* value: .006) to have an increased risk in multivariate models. Patients with CD4 counts >100 cells/μL at baseline had reduced risks of developing sustained ALT elevations >40 IU/L (CD4 count 100-200 cells/μL: HR: 0.79; 95% CI, 0.67-0.95; *P* value = .01, and CD4 count >200 cells/μL: HR: 0.70; 95% CI, 0.55-0.89; *P* value = .004). There also appeared to be an increased risk of sustained ALT elevations >40 IU/L among male patients, those initiated on d4T+3TC+NVP, and those receiving multiple RDA, though these were not statistically significant (Table 2).

**Table 2.** Risk Factors for Incident Mild or Moderate ALT Elevation (>40 IU/L) and Sustained Mild or Moderate ALT Elevation (>40 IU/L at 2 or More Consecutive Visits).

Variable	Mild or Moderate ALT Elevation (ALT >40 IU/L)					Sustained Mild or Moderate ALT Elevation (ALT >40 IU/L at 2+ Consecutive Visits)				
	n/N (%)	Univariate		Multivariate		n/N (%)	Univariate		Multivariate	
		Hazard Ratio (95% CI)	*P* Value	Hazard Ratio (95% CI)	*P* Value		Hazard Ratio (95% CI)	*P* Value	Hazard Ratio (95% CI)	*P* Value
Sex										
Female	785/1873 (41.9)	Ref		Ref		388/817 (47.5)	Ref			Ref
Male	410/794 (51.6)	1.40 (1.24-1.58)	<.001	1.44 (1.27-1.64)	<.001	233/422 (55.2)	1.15 (0.98-1.35)	.09	1.18 (0.99-1.40)	.07
Age, years										
≤30	165/414 (39.8)	Ref		Ref		73/171 (42.7)	Ref		Ref	
31-45	796/1719 (46.3)	1.18 (0.99-1.39)	.05	1.15 (0.97-1.37)	.09	431/828 (52.1)	1.22 (0.95-1.56)	.12	1.21 (0.94-1.55)	.15
>45	232/528 (43.9)	1.10 (0.90-1.35)	.33	1.03 (0.84-1.27)	.74	117/238 (49.2)	1.07 (0.80-1.44)	.62	1.03 (0.76-1.39)	.85
WHO HIV disease stage										
I or II	282/585 (48.2)	Ref		Ref		175/291 (60.1)	Ref		Ref	
III	717/1615 (44.4)	0.91 (0.79-1.05)	.19	0.94 (0.82-1.09)	.44	347/741 (46.8)	0.75 (0.63-0.90)	.002	0.76 (0.63-0.93)	.006
IV	116/274 (42.3)	0.89 (0.79-1.22)	.89	0.92 (0.73-1.15)	.45	60/125 (48.0)	0.89 (0.67-1.20)	.47	0.89 (0.65-1.23)	.49
CD4 count, cells/μL										
<100	503/1032 (48.7)	Ref		Ref		274/515 (53.2)	Ref		Ref	
100-200	438/994 (44.1)	0.79 (0.70-0.91)	.001	0.81 (0.71-0.92)	.002	234/459 (50.9)	0.79 (0.67-0.95)	.01	0.79 (0.66-0.94)	.01
>200	222/566 (39.2)	0.70 (0.59-0.82)	<.001	0.74 (0.63-0.87)	<.001	101/229 (44.1)	0.67 (0.52-0.83)	<.001	0.70 (0.55-0.89)	.004
HIV viral load, copies/mL										
<100 000	157/357 (43.9)	Ref		Ref		90/162 (55.6)	Ref		Ref	
100 000-1 000 000	337/730 (46.2)	1.07 (0.88-1.29)	.49	1.01 (0.83-1.22)	.91	181/350 (51.7)	0.88 (0.68-1.14)	.34	0.83 (0.64-1.08)	.16
>1 000 000	24/60 (40.0)	0.95 (0.62-1.46)	.82	0.82 (0.53-1.27)	.37	10/24 (41.7)	0.79 (0.41-1.52)	.48	0.71 (0.36-1.41)	.33
cART regimen										
AZT+3TC+EFV	114/271 (42.1)	Ref		Ref		60/122 (49.2)	Ref		Ref	
d4T+3TC+NVP	768/1629 (47.2)	1.29 (1.05-1.57)	.01	1.44 (1.17-1.76)	<.001	398/794 (50.1)	1.34 (1.01-1.75)	.04	1.32 (0.99-1.75)	.056
d4T+3TC+EFV	99/248 (39.9)	1.03 (0.79-1.35)	.82	1.09 (0.83-1.44)	.51	53/104 (50.9)	1.21 (0.83-1.74)	.32	1.17 (0.81-1.71)	.36
AZT+3TC+NVP	89/223 (39.9)	0.99 (0.75-1.30)	0.93	1.06 (0.80-1.41)	0.67	44/89 (49.4)	1.16 (0.79-1.72)	0.44	1.16 (0.77-1.74)	0.46
BMI groups, kg/m^2^										
<18.5	269/601 (44.8)	Ref		Ref		137/275 (49.8)	Ref		Ref	
18.5-25	640/1476 (43.4)	0.89 (0.75-0.99)	.05	0.89 (0.77-1.03)	.12	319/663 (48.1)	0.86 (0.70-1.05)	.14	0.86 (0.69-1.07)	.17
>25	212/414 (51.2)	1.02 (0.86-1.23)	.77	1.05 (0.87-1.27)	.60	128/225 (56.9)	0.94 (0.74-1.20)	.65	0.93 (0.71-1.21)	.59
Hemoglobin, g/dL										
≥8.5	915/1999 (45.8)	Ref		Ref		491/948 (51.8)	Ref		Ref	
<8.5-severe anemia	236/576 (40.9)	0.94 (0.81-1.08)	.38	0.97 (0.83-1.12)	.66	115/247 (46.6)	1.09 (0.89-1.34)	.39	1.09 (0.88-1.35)	.42
Self-reported alcohol intake										
No	1110/2434 (45.6)	Ref		Ref		588/1154 (50.9)	Ref		Ref	
Yes	25/45 (55.6)	1.26 (0.85-1.87)	.25	1.17 (0.78-1.74)	.45	13/25 (52.0)	0.86 (0.49-1.49)	.60	0.84 (0.48-1.47)	.54
TB coinfection										
No	1183/2640 (44.8)	Ref		Ref		616/1227 (50.2)	Ref		Ref	
Yes	12/27 (44.4)	1.03 (0.58-1.82)	.92	1.19 (0.66-2.13)	.56	5/12 (41.7)	0.72 (0.29-1.73)	.46	0.86 (0.35-2.13)	.74
Hepatitis B										
Negative	761/1726 (44.1)	Ref		Ref		392/787 (49.8)	Ref		Ref	
Positive	48/99 (48.5)	1.17 (0.87-1.56)	.29	1.04 (0.78-1.41)	.76	28/53 (52.8)	1.06 (0.72-1.55)	.77	0.96 (0.65-1.42)	.83
Hepatitis C										
Negative	610/1433 (42.6)	Ref		Ref		298/636 (46.9)	Ref		Ref	
Positive	16/25 (64.0)	1.71 (1.04-2.81)	.04	1.64 (0.99-2.71)	.05	7/16 (43.7)	0.88 (0.42-1.86)	.74	0.79 (0.37-1.71)	.56
Total cholesterol, mg/dL										
<200	814/1828 (44.5)	Ref		Ref		423/837 (50.5)	Ref		Ref	
≥200	116/235 (49.4)	1.09 (0.90-1.33)	.36	1.09 (0.89-1.33)	.40	69/120 (57.5)	1.07 (0.83-1.38)	.58	1.09 (0.84-1.41)	.53
Triglycerides, mg/dL										
<150	715/1628 (43.9)	Ref		Ref		373/737 (50.6)	Ref		Ref	
≥150	195/386 (50.5)	1.31 (1.12-1.54)	.001	1.26 (1.07-1.48)	.01	103/200 (51.5)	1.08 (0.87-1.34)	.49	1.07 (0.85-1.34)	.55
Randomized multivitamin regimen										
Single RDA	524/1337 (39.2)	Ref		Ref		258/543 (47.5)	Ref		Ref	
Multiple RDA	671/1330 (50.1)	1.41 (1.26-1.58)	<.001	1.40 (1.25-1.57)	<.001	363/696 (52.2)	1.19 (1.04-1.36)	.01	1.15 (0.98-1.36)	.08

Abbreviations: ALT, alanine aminotransferase; AZT, zidovudine; BMI, body mass index; cART, combination antiretroviral therapy; CI, confidence interval; d4T, stavudine; EFV, efavirenz; NVP, nevirapine; RDA, recommended dietary allowances; TB, tuberculosis; WHO, World Health Organization; 3TC, lamivudine.

Predictors of severe ALT elevations >200 IU/L are also presented in Table 3. In univariate models, male sex, self-reported alcohol consumption, and hepatitis B coinfection were significant predictors of severe ALT elevations (*P* values <.05). In multivariate models, individuals with hepatitis coinfection remained at significantly increased risk of incident ALT >200 IU/L (HR: 2.50; 95% CI, 1.16-5.40; *P* value: 0.02). In addition, patients who reported alcohol consumption were at 3.08 (95% CI, 1.20-7.92; *P* value: .02) times the risk of incident ALT >200 IU/L when compared to those who did not. In addition, patients with hepatitis C coinfection also appeared to be at high risk of severe ALT elevations >200 IU/L, but results did not reach statistical significance (HR: 3.75; 95% CI, 0.83-16.96; *P* value: .08).

**Table 3.** Risk Factors for Incident Severe ALT Elevation (>200 IU/mL).

Variable	Severe ALT Elevation (ALT >200 IU/L)				
	n/N (%)	Univariate		Multivariate	
		Hazard Ratio (95% CI)	*P* Value	Hazard Ratio (95% CI)	*P* Value
Sex					
Female	43/2063 (2.1)	Ref		Ref	
Male	32/957 (3.3)	1.68 (1.06-2.65)	.03	1.47 (0.90-2.41)	.12
Age, years					
≤30	7/463 (1.5)			Ref	
31-45	56/1959 (2.9)	1.87 (0.85-4.10)	.12	1.62 (0.73-3.59)	.24
>45	12/592 (2.0)	1.33 (0.52-3.39)	.54	1.29 (0.49-3.36)	.59
WHO HIV disease stage					
I or II	18/650 (2.8)	Ref		Ref	
III	44/1802 (2.4)	0.91 (0.52-1.57)	.73	0.89 (0.49-1.61)	.71
IV	8/338 (2.4)	0.95 (0.41-2.19)	.91	0.79 (0.32-1.96)	.62
CD4 count, cells/μL					
<100	29/1212 (2.4)	Ref		Ref	
100-200	27/1111 (2.4)	0.95 (0.56-1.59)	.84	0.98 (0.57-1.69)	.96
>200	16/608 (2.6)	1.05 (0.57-1.93)	.88	1.14 (0.60-2.15)	.69
HIV viral load, copies/mL					
<100 000	14/386 (3.6)	Ref		Ref	
100 000-1 000 000	22/829 (2.6)	0.73 (0.37-1.42)	.35	0.65 (0.33-1.31)	.23
>1 000 000				–	
cART regimen					
AZT+3TC+EFV	13/344 (3.8)	Ref		Ref	
d4T+3TC+NVP	46/1785 (2.6)	0.69 (0.38-1.29)	.25	0.79 (0.42-1.51)	.49
d4T+3TC+EFV	4/299 (1.3)	0.37 (0.12-1.13)	.08	0.36 (0.11-1.12)	.08
AZT+3TC+NVP	4/249 (1.6)	0.42 (0.14-1.31)	.14	0.45 (0.14-1.39)	.17
BMI groups, kg/m^2^					
<18.5	21/704 (2.9)	Ref		Ref	
18.5-25	37/1659 (2.2)	0.68 (0.40-1.17)	.17	0.63 (0.36-1.09)	.10
>25	13/449 (2.9)	0.87 (0.43-1.73)	.69	0.76 (0.36-1.61)	.47
Hemoglobin, g/dL					
≥8.5	59/2277 (2.6)	Ref		Ref	
<8.5-severe anemia	14/638 (2.2)	0.91 (0.51-1.63)	.74	1.02 (0.55-1.91)	.94
Self-reported alcohol intake					
No	69/2743 (2.5)	Ref		Ref	
Yes	5/52 (9.6)	4.0 (1.61-9.92)	.003	3.08 (1.20-7.92)	.02
TB coinfection					
No	75/2998 (2.5)	Ref		Ref	
Yes	–	–		–	–
Hepatitis B					
Negative	47/1925 (2.4)	Ref		Ref	
Positive	8/127 (6.3)	2.67 (1.26-5.65)	.01	2.50 (1.16-5.40)	.02
Hepatitis C					
Negative	33/1591 (2.1)	Ref		Ref	
Positive	2/27 (7.4)	3.46 (0.83-14.4)	.09	3.75 (0.83-16.96)	.08
Total cholesterol, mg/dL					
<200	47/2049 (2.3)	Ref		Ref	
≥200	5/272 (1.8)	0.77 (0.31-1.95)	.59	0.67 (0.26-1.73)	.41
Triglycerides, mg/dL					
<150	37/1794 (2.1)	Ref		Ref	
≥150	14/472 (2.9)	1.57 (0.85-2.91)	.15	1.74 (0.93-3.28)	.08
Randomized multivitamin regimen					
Single RDA	31/1521 (2.0)	Ref		Ref	
Multiple RDA	44/1499 (2.9)	1.44 (0.91-2.28)	.12	1.38 (0.87-2.21)	.17

Abbreviations: ALT, alanine aminotransferase; AZT, zidovudine; BMI, body mass index; cART, combination antiretroviral therapy; CI, confidence interval; d4T, stavudine; EFV, efavirenz; NVP, nevirapine; RDA, recommended dietary allowances; TB, tuberculosis; WHO, World Health Organization; 3TC, lamivudine.

## Discussion

In this prospective cohort study of HIV-infected Tanzanian adults initiating cART, we found that approximately half of patients had at least 1 incident ALT elevation of >40 IU/L with about one-quarter experiencing a sustained ALT elevation. Male sex, CD4 count <100 cells/μL, triglyceride concentrations ≥150 mg/dL, and d4T+3TC+NVP cART were found to be associated with increased risk of ALT elevations >40 IU/L. Incident severe ALT elevations >200 IU/L occurred in 2.5% of patients and risk factors included hepatitis B coinfection and alcohol consumption.

The interpretation and clinical significance of ALT elevations is complex, since liver enzyme abnormalities can be the result of multiple factors including the antiretroviral drugs, hepatitis and other viral coinfections, and other causes of liver disease such as steatosis and hepatotoxicity related to alcohol and use of illicit drugs. Some of these factors also commonly co-occur in HIV-infected populations, which can further complicate clinical management.^7^ Thus, clinicians should be aware of the frequency and severity of elevated liver enzymes and need to thoroughly investigate the patient history, drug levels as well as monitor immunological and virological biomarkers to elucidate potential causes and the need for intervention. Severe liver enzyme elevations (ALT >200) warrants clinical intervention due to the risk of pathological liver disease and liver fibrosis.^20^

We found that 48% of individuals had mild-to-moderate ALT elevations (>40 IU/L), with a little over half of them having persistently elevated ALT levels; the risk of incident severe ALT elevation >200 was low (2.5%). Although 2.5% of the patients presented with ALT >200 IU/L, none presented with any clinically relevant features suggestive of hepatotoxicity that warranted any intervention. The incidence of elevated ALT was greater in our study when compared to studies in developed countries (<30%),^21,22^ but similar with studies conducted from Sub-Saharan Africa.^23,24^ A previous cross-sectional study of ART-naive Tanzanian adults a found 13% prevalence of ALT >40 IU/L and 0.3% for ALT >200 IU/L.^25^ As a result, antiretroviral use appears to be a key contributor to high rates of liver enzyme elevations.

Most classes of antiretroviral drugs have been shown to increase the risk of liver enzyme abnormalities.^7,11,26^ The most frequently implicated antiretroviral groups are the NNRTIs followed by protease inhibitors and the NRTIs).^13^ Common culprits are NVP (an NNRTI) and d4T (NRTI), which were the first-line antiretrovirals used by two-thirds of our study population.^10,27^ We also determined that the risk of ALT elevation >40 IU/L was 60% higher for individuals who received both d4T and NVP. Nevertheless, both d4T and NVP are no longer used as first-line cART drugs in Tanzania and most HIV treatment programs in Sub-Saharan Africa.

In addition, we found that lower CD4 counts at cART initiation was associated with increased risk of ALT >40 IU/L. The direction of the relationship between CD4 count and ALT elevations has not been consistently reported. Sulkowski et al reported that a greater increase in CD4 count was associated with increased risk of severe hepatotoxicity (ALT >200 IU/L) in HIV–hepatitis coinfection.^28,29^ In studies of HIV mono-infected patients that excluded viral hepatitis, one reported an increased risk of ALT with lower CD4, while another study reported the opposite findings.^20,22^

We also found that hepatitis B was an independent predictor of developing ALT elevations >200 IU. Chronic hepatitis increases the risk of developing hepatotoxicity, particularly in patients receiving NVP.^13,30,31^ Studies have documented a flare in hepatotoxicity at 4 to 6 months after ART initiation, which is consistent with our findings, as the majority of incident ALT elevations were detected at the first 4-month assessment. Alcohol abuse is also a well-established risk factor for ALT elevations.^20^ In a study by Pol et al, HIV-infected patients who consumed <40 g/d of alcohol were at risk of alcohol-associated hepatitis, suggesting that HIV-infected patients may be more sensitive to alcohol toxicity.^7^ This is consistent with our finding that individuals who consumed alcohol had 3 times the risk of severe ALT elevations.

Results from our study have shown that elevated serum triglyceride level to be a predictor for ALT elevations. We found that triglyceride levels >150 mg/dL at cART initiation increased the risk of ALT incident ALT >40 and >200 IU/L. Accumulation of triglycerides in the liver can result in non-alcoholic fatty liver disease (NAFLD) and may present with varying degrees of liver enzyme abnormalities.^32^ Evidence suggests that in untreated HIV infection, the absence of cART-related toxicity may have direct impact on liver dysfunction, including NAFLD and NASH.^6,33^ Nonalcoholic steatohepatitis has been reported to increase the risk of liver enzyme abnormalities among HIV-infected patients.^6^ As a result, assessment of triglycerides at ART initiation can identify patients at increased risk of ALT elevations.

As reported in the parent trial, patients randomized to multiple RDA multivitamins had increased risk of incident ALT >40 IU/L. We further examined this question and noted that multiple RDA multivitamins were not associated with a statistically significant persistent increase in the risk of sustained ALT >40 IUL elevation or an increase the risk of ALT >200 IU/L. The mechanism by which multiple RDA multivitamins may increase the risk of ALT elevations remains unclear. A recent implementation evaluation of a routine multivitamin supplementation program for adults enrolled in Dar es Salaam HIV care and treatment program determined that single-dose RDA multivitamins for adults on ART and multiple RDA multivitamins for ART-naïve patients were both associated with decreased risk of ALT elevations >40 IU/L.^34^ Patients with varying degrees of liver dysfunction have increased oxidative stress, and it has been postulated that supplementation with vitamins E and C may counteract oxidative stress.^35,36^ Nevertheless, there have been some reports that suggested higher rates of liver toxicity with higher doses of vitamin B, particularly niacin (vitamin B_3_) that was used for treatment of various conditions such as hypercholesterolemia and skin diseases.^37,38^

Our study had several limitations. First, due to the observational design of the study, we cannot rule out residual or unmeasured confounding. We did not collect data on drug use, cholesterol levels, diabetes, and other factors, which may be associated with ALT elevations. In addition, alcohol and cigarette use were self-reported, which likely lead to misclassification that likely attenuated any association. This study did not use other laboratory markers of liver damage such as aspartate aminotransferase, serum glutamic-oxaloacetic transaminase, albumin, or bilirubin. In addition, ALT is an insensitive marker for long-term liver damage, and therefore future research should also use more specific test for liver disease (ie, Fibroscan). At the time the study was conducted, NVP was the default NNRTI backbone in ART. At present, d4T and NVP are no longer part of the treatment regimens recommended by WHO and Tanzania. Implications from this study show that NNRTIs and, more so, NVP may be associated with liver toxicity. Currently, the most recent regimens exclude the use of NNRTIs and instead use a combination of 2NRTI plus 1 integrase inhibitor, thus reducing the risk of hepatotoxicity.

Overall, we determined that incident mild-to-moderate ALT elevations (>40 IU/L) occurred in nearly half of the study and 2.5% of patients had severe ALT elevations (>200 IU/L). Predictors of ALT elevations >40 IU/L included male sex, d4T+3TC+NVP cART regimen, low CD4 counts, and high serum triglyceride levels. Hepatitis B coinfection and alcohol consumption increased the risk of incident severe ALT elevations >200 IU/L. Future research should focus on strategies to mitigate the risk of liver enzyme abnormalities and liver disease among HIV-infected adults initiating ART in Tanzania and other resource-limited settings.
